# No association between delayed graft function and BK polyomavirus infection reactivation after kidney transplantation: a systematic review and meta-analysis

**DOI:** 10.3389/fneph.2026.1790723

**Published:** 2026-06-23

**Authors:** Thidarat Kitrungphaiboon, Felicity Evison, Suzy Gallier, Adnan Sharif

**Affiliations:** 1 Department of Nephrology, Bhumirajanagarindra Kidney Institute Hospital, Bangkok, Thailand; 2 Statistics Team: Research, Development and Innovation, University Hospital Birmingham, Birmingham, United Kingdom; 3 Department of Nephrology and Transplantation, University Hospitals Birmingham, Birmingham, United Kingdom; 4 Institute of Immunology and Immunotherapy, University of Birmingham, Birmingham, United Kingdom

**Keywords:** BK virus, BKPyV, BKPyVAN, BKPyV DNAemia, delayed graft function, kidney transplantation, meta-analysis, polyoma virus

## Abstract

**Introduction:**

An association between delayed graft function (DGF) and BK polyomavirus (BKPyV) DNAemia is not well defined.

**Methods:**

We investigated this with 1) a retrospective review of a single-center cohort analysis, and 2) a systematic review & meta-analysis of published data. Kidney transplant recipients at a single-center between 01/01/2007-30/06/2018 were analyzed. Comparative analyses were done with logistic regression models based upon clinical risk factors. Following a systematic review of published studies, meta-analysis was performed using the DerSimonian-Laird random effects model.

**Results:**

In our single-center analysis, we analyzed 1,770 kidney transplant recipients with median follow up 5.3 years (interquartile range 2.7-8.7 years). BKPyVDNAemia was associated with; male sex (8.3% versus 5.3% respectively, p=0.017), ABO-incompatible transplantation (10.3% versus 4.6% respectively, p=0.004) and DGF (9.0% versus 6.3% respectively, p=0.048). In a multivariate analysis, only recipient male sex and ABO-incompatible transplantation was associated with BKPyVDNAemia. In a systematic review of published literature, we identified 11 studies and performed a meta-analysis of empirical data including our Birmingham cohort data. No significant association was observed between DGF rates and BKPyVDNAemia (OR 1.00, 95% CI 0.67-1.50).

**Discussion:**

We did not identify any association between DGF rates and subsequent BKPyVDNAemia.

## Background

Delayed graft function (DGF) is a common early complication after kidney transplantation, clinically defined as the need for dialysis within the first postoperative week. Due to evolving deceased donor characteristics (e.g., increasing age), longer cold ischemic times, and the expanding use of donors after circulatory death (DCD), DGF rates are significant in contemporary clinical practice. After deceased donor kidney transplant (DDKT), the incidence of DGF is estimated to range between 20%–45% (1–3), while living kidney transplantation has a lower DGF incidence of approximately 1.6%–3.6% (4–6).

One of the main reasons for this difference in DGF rates between deceased- and living-donor transplantation relates to the risk of pathophysiological insult from ischemia-reperfusion injury (IRI), which is less pronounced with living-donor transplantation. Ischemia is associated with adenosine triphosphate (ATP) depletion, endothelial cell injury, and prolonged ischemia that subsequently proceeds to reperfusion injury-induced generation of reactive oxygen species, activation of the complement system, and cytokine release (7, 8). While DGF is the immediate clinical manifestation of IRI, the direct and indirect clinical sequelae of DGF include an increased risk for allograft rejection (9, 10), lower kidney function and allograft survival (10–13), prolonged hospitalization (14), and increased patient mortality (2, 10).

Emerging reports speculate on an association between DGF and an increased risk for infection (15, 16), which may relate to pathophysiological changes that create an environment susceptible to infection. An opportunistic infection that could thrive in the DGF environment is BK polyomavirus (BKPyV) infection, which can lead to BK polymavirus DNAemia (BKPyV DNAemia) and/or BK polyomavirus associated nephropathy (BKPyVAN). BKPyV is a significant complication after kidney transplantation, observed in up to 30% of kidney transplant recipients (17), and is associated with allograft dysfunction and graft loss (18, 19). BKPyV commonly occurs in the early phase of kidney transplantation, usually within the first 2–6 months after surgery (20). This early onset could be due to several factors, including the burden of immunosuppression and urothelial trauma from stent insertions (21). IRI could be another pathophysiological factor associated with BKPyV infection (22). BKPyV primarily replicates in renal tubular epithelial cells and urothelial cells, which can be damaged by IRI, leading to increased susceptibility. IRI may also create an inflammatory microenvironment through the release of pro-inflammatory cytokines (e.g., interleukin-6, tumor necrosis factor-α) that promotes viral transcription from latency. This local inflammation and tissue injury from IRI may impair T-cell surveillance, allowing BKPyV to replicate unchecked. Combined with heightened immunosuppression in this early post-surgery phase to avoid rejection, a pro-inflammatory microenvironment with reduced immunosurveillance indicates a plausible association linking DGF with the risk for BKPyV infection.

However, clinical reports showing an association between DGF and BKPyV infection yield conflicting results. While some narrative reviews discussing risk factors (23) cite an association between DGF and BKPyV, the latest consensus meeting report did not identify DGF as a BKPyV infection risk factor (24). Several observational studies have reported higher rates of BKPyV DNAemia or BKPyVAN among recipients experiencing DGF, hypothesizing that IRI and the subsequent immunologic and inflammatory milieu may facilitate viral reactivation (25–29). Conversely, other studies have failed to demonstrate a clear link, citing confounding factors such as variations in immunosuppression intensity and donor quality (16, 30). This inconsistency in the literature reflects ongoing clinical equipoise. While transplant clinicians often view DGF and BKPyV infection as independent complications, the possibility of a causal or contributory relationship between them remains unresolved. A clearer understanding of this association could inform more nuanced risk stratification and post-transplant management strategies, particularly in high-risk recipients.

Therefore, this study aimed to investigate this association through (1) a retrospective review of a single-center cohort and (2) a systematic review and meta-analysis of published studies. By synthesizing the current evidence on the relationship between DGF and BKPyV infection, knowledge gaps may be identified that warrant future investigation.

## Materials and methods

### Study population

We performed a retrospective cohort study and analyzed all kidney transplant procedures between 1 January 2007 and 30 June 2018, at a single transplant center (University Hospitals Birmingham, UK). We excluded recipients of multiple organ transplants.

### Data sources

Local data were electronically extracted by the hospital informatics team for every patient, with manual data linkage to electronic patient records for classification of both DGF and BKPyV infection. All data were collected from electronic patient records unless otherwise stated. Acute rejection, 1-year creatinine, and patient and graft survival data were acquired and linked from NHS Blood & Transplant. Hospitalization data were acquired from Hospital Episode Statistics, an administrative data warehouse containing admissions to all National Health Service hospitals in the UK. It contains detailed records relating to individual patient treatments, with data extraction facilitated by utilizing codes on procedural classifications [Office of Population Censuses and Surveys Classification of Interventions and Procedures, 4th revision (OPCS-4)] and medical classifications [World Health Organization International Classification of Disease, 10th revision ICD-10)].

### Diagnostic criteria for DGF and BKPyV

DGF was defined as the need for dialysis within the first post-operative week after surgery, while BKPyV DNAemia was defined as >200 copies/mL on two consecutive visits. During the study period, BKPyV DNAemia was not systematically screened and was checked only in the context of transplant dysfunction. More recently, a systematic BKPyV DNAemia screening program within the first 2 years after kidney transplantation has been implemented.

### Immunosuppression protocol

All patients received the same immunosuppression over the study period, with minimization of tacrolimus exposure in line with the SYMPHONY protocol (31). Induction therapy was with basiliximab (20 mg on days 0 and 4) for standard-risk immunological patients or alemtuzumab (30 mg on day 0) for high-risk immunological patients. All patients received methylprednisolone (500 mg on day 0). Maintenance therapy included tacrolimus (target 12-h trough levels 5 – 8 ng/L), mycophenolate mofetil (MMF, 2 g daily with tapering to 1 g daily after 6 months), and maintenance corticosteroids (20 mg daily, weaned down to 5 mg daily by 3 months). Biopsies were indication-based in the context of transplant dysfunction (categorized as ≥20% creatinine rise or new-onset proteinuria). Biopsy data were classified in accordance with Banff criteria (32).

Episodes of acute cellular rejection were treated with a bolus of corticosteroids, with T-cell depletion therapy (alemtuzumab) for steroid-resistant rejection. Antibody-mediated rejection was treated with antibody removal by plasmapheresis ± intravenous immunoglobulin. Viral serology (e.g., BKPyV DNAemia) and/or anti-HLA antibodies were checked on an indication basis, based on transplant dysfunction.

### Outcomes

The primary outcome measure was the development of BKPyV DNAemia after kidney transplantation.

### Statistical analysis

Initially, a range of demographic and transplant characteristics were compared between the follow-up groups. Normality of data was assessed using the Kolmogorov-Smirnov tests. Descriptive statistics were used to estimate frequencies. Categorical variables are presented as number (%) and continuous variables as mean (± standard deviation) or median (± interquartile range), depending upon the normality of distribution. Differences between groups were assessed with χ² or two-sided Fisher’s exact test for categorical variables and the *t*-test or Mann–Whitney *U* test to compare continuous variables.

Survival was analyzed as the time from placement on the waiting list to death, with data censored at loss of follow-up or on 31 December 2020. Time-to-graft-loss models were conducted using survival/censoring-weighted Cox regression and adjusted for age, sex, ethnicity, cause of kidney failure (diabetes or not), level of HLA mismatches, ABO-incompatible transplantation, DGF, and rejection. Analyses were performed based on a positive BKPyV DNAemia result (defined as >200 copies/mL), with a sub-analysis based on a clinically meaningful BKPyV DNAemia result (defined as >5,000 copies/mL). A *p*-value less than 0.05 was considered significant, and less than 0.001 was considered highly significant. Statistical analyses were undertaken using R (version 4.3.2).

Assuming a BKPyV DNAemia rate of approximately 12% (as per British Transplantation Society guidelines) (33)) a sample size of 2,036 per arm would provide 80% power to demonstrate the absence of a meaningful association (assuming a minimum effect size of 25% and two-sided alpha = 0.05). Changing the equivalence margin effect size to 15% would require a sample size of 5,444 per arm. As this cohort size would not be available in our single-center cohort, a meta-analysis of empirical data from published studies was planned.

### Systematic review and meta-analysis

Two investigators (TK and AS) independently searched published studies indexed in MEDLINE, EMBASE, and the Cochrane database from inception through September 2024. Search terms included (“Delayed Graft Function” [MeSH] OR “delayed graft function” [tiab] OR DGF [tiab]) AND (“BK virus” [MeSH] OR “BK virus” [tiab] OR “BK polyomavirus” [tiab]). Additionally, a manual search was performed for relevant studies using references from all retrieved articles. The primary data extracted from each study included all BKPyV events and total numbers for each patient cohort, stratified by available DGF status. No language restrictions were applied. This study is reported in accordance with the MOOSE checklist for reporting meta-analyses of observational studies (34).

Study eligibility was independently determined by the two investigators, with any discrepancy resolved by mutual consensus. We conducted a meta-analysis utilizing all eligible studies identified in our systematic review, including our single-center data. Given the high likelihood of inter-study variances, we used a random-effects model rather than a fixed effect model to determine effect size by odds ratio. Statistical heterogeneity was assessed using the I2 statistic, which quantifies the proportion of the total variation across studies due to heterogeneity rather than chance. An I2 value of 0% to 25% represents insignificant heterogeneity, 26% to 50% low heterogeneity, 51% to 75% moderate heterogeneity, and >75% high heterogeneity. Subgroup analyses and meta-regression techniques were used to investigate any significant heterogeneity. Meta-analysis was performed using the DerSimonian–Laird random effects model using R (version 4.3.2).

### Approvals

This study received institutional review board approval and was registered as an audit (audit identifier: CARMS-12578). The corresponding author had full access to all data. The data that support the findings of this study are available from the corresponding author upon reasonable request.

## Results

### Study cohort

Data were analyzed for 1,770 kidney transplant recipients, with a median follow-up of 5.3 years (IQR 2.7 to 8.7 years). The cohort demographics were 59.8% male, with a median age of 48 ± 21 years, and 64.7% white ethnicity. In total, 72.5% of kidney transplant recipients received deceased donor kidneys. The most common cause of end-stage kidney failure was glomerulonephritis (17.7%), followed by diabetes (12.2%). Repeat kidney transplant recipients constituted 7.2% of the study cohort. Our cohort included 88 procedures involving ABO-incompatible kidney transplantation (5.0%).

### Delayed graft function

DGF was observed in 31.1% of kidney transplant recipients post-surgery, with most cases (96.3%) occurring in the context of DDKT.

### BKPyV infection

One hundred twenty-six kidney transplant recipients (7.1%) developed BKPyV DNAemia post-transplantation. Of these patients, 34.9% had biopsy-proven BKPyVAN. The median time to development of BKPyV DNAemia was 242 ± 101 days.

**Table 1** outlines the unadjusted factors associated with the development of BKPyV DNAemia. BKPyV DNAemia was associated with male sex (8.3% versus 5.3% without BKPyV DNAemia, p = 0.017), ABO-incompatible transplantation (10.3% versus 4.6%, p = 0.004), and delayed graft function (9.0% versus 6.3%, p = 0.048). In an adjusted logistic regression model (**Table 2**), only male sex and ABO-incompatible kidney transplantation were identified as independent risk factors for BKPyV DNAemia. In a sub-analysis using a BKPyV DNAemia threshold of 5,000 copies/mL (n = 109), only male sex was identified as a risk factor for BKPyV DNAemia (OR for female sex: 0.58; 95% CI 0.29–0.87, p = 0.031).

**Table 1:** Demographics stratifying kidney transplant recipients with and without BKPyV DNAemia.

Variable		BKPyV DNAemia	No BKPyV DNAemia	P value
Detected BKPyV DNAemia (n)		126	1,644	–
Age (mean ± SD) in years		48.8 ± 14.4	46.6 ± 13.8	0.087
Deceased donor		99 (78.6%)	1,184 (72.0%)	0.683
Sex	Male	88 (8.3%)	970 (91.7%)	0.017
	Female	38 (5.3%)	674 (94.7%)	
Ethnicity	White	74 (58.7%)	1,072 (65.2%)	0.234
	Black	6 (4.8%)	95 (5.8%)	
	South Asian	27 (21.4%)	328 (20.0%)	
	Other	14 (11.1%)	110 (6.7%)	
	Unknown	5 (4.0%)	39 (2.4%)	
ABO incompatible transplant		13 (10.3%)	75 (4.6%)	0.004
Pre-existing diabetes		12 (9.8%)	150 (9.5%)	0.911
HLA-A mismatch	0	22 (18.2%)	372 (23.5%)	0.358
	1	64 (52.9%)	752 (47.5%)	
	2	35(28.9%)	458 (29.0%)	
HLA-B mismatch	0	21 (17.4%)	296 (18.7%)	0.593
	1	79 (65.3%)	1,064 (67.3%)	
	2	21 (17.4%)	222 (14.0%)	
HLA-DR mismatch	0	58 (47.9%)	699 (44.2%)	0.554
	1	53 (43.8%)	772 (48.8%)	
	2	10 (8.3%)	111 (7.0%)	
Cold ischemic time (min)		669 ± 482	741 ± 488	0.213
Calculated reaction frequency* (%)		15 ± 6	18 ± 7	0.305
Induction	Basiliximab	122 (96.8)	1,599 (97.3%)	0.983
	Alemtuzumab	4 (3.2%)	45 (2.7%)	
Mean (± SD) tacrolimus level within the first 3 months (ug/mL)		7.6 ± 4.6	6.9 ± 3.2	0.576
Mean (± SD) MMF dose per day within the first 3 months (mg)		1,870	1,722	0.700
Maintenance steroids		124 (98.4%)	1,631 (99.2%)	0.892
Delayed graft function		48 (9.0%)	78 (6.3%)	0.048
Any rejection within 3 months		10 (7.6%)	90 (6.8%)	0.745
Any urological complication within 1-year**		6 (4.8%)	69 (4.2%)	0.749

*Equivalent to calculated panel reactive antibody.

**Defined as urine leak, any urological intervention or any urological surgery (routine planned procedures excluded, e.g., ureteric stent removal).

**Table 2:** Logistic regression model for predictors of BKPyV DNAemia.

Variable		Odds ratio	95% CI	P value
Age in years		1.01	0.99–1.03	0.421
Deceased donor		1.18	0.98–1.42	0.083
Sex	Male	REF		0.008
	Female	0.49	0.33–0.78	
Ethnicity	White	REF		
	Black	0.87	0.30–2.10	0.832
	South Asian	1.06	0.52–2.05	0.745
	Other	1.46	0.77–3.51	0.833
	Unknown	2.67	0.79–8.01	0.091
ABO incompatible transplant		3.95	1.83–7.77	<0.001
Pre-existing diabetes		1.05	0.62–1.72	0.928
HLA-A mismatch	0	REF		
	1	1.67	0.47–2.32	0.204
	2	1.47	0.48–2.70	0.378
HLA-B mismatch	0	REF		
	1	1.11	0.89–2.34	0.298
	2	1.43	0.65–2.80	0.536
HLA-DR mismatch	0	REF		
	1	0.65	0.54–2.08	0.592
	2	0.88	0.56–2.98	0.401
Cold ischemic time (minutes)		1.67	0.87–2.65	0.219
Calculated reaction frequency* (%)		1.20	0.78–1.76	0.254
Induction	Basiliximab	REF		0.107
	Alemtuzumab	1.46	0.88–2.24
Mean (± SD) tacrolimus level within the first 3 months (ug/mL)		1.55	0.91–1.98	0.098
Mean (± SD) MMF dose per day within the first 3 months (mg)		1.34	0.82–1.72	0.428
Maintenance steroids				
Delayed graft function		1.33	0.65–2.38	0.145
Any rejection within 3 months		0.92	0.46–1.62	0.641
Any urological complication within 1-year**		1.25	0.34–5.76	0.732

### Outcomes after BKPyV DNAemia

**Figure 1:**
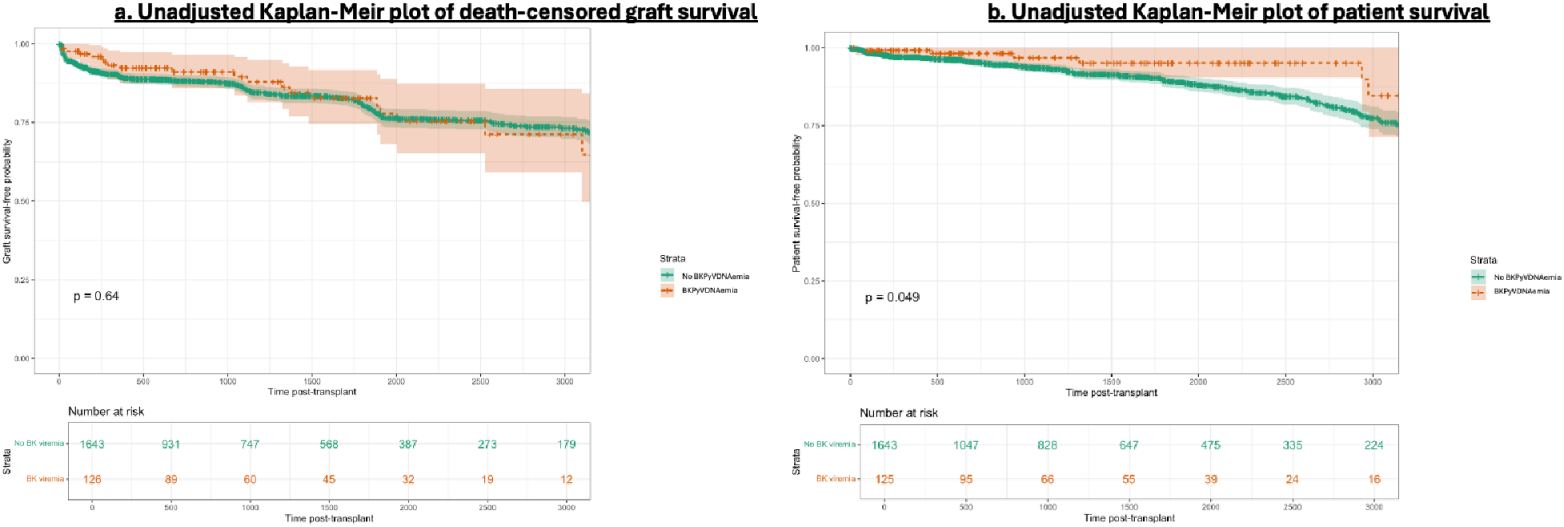
Unadjusted Kaplan–Meir plot of **(A)** death-censored graft survival and **(B)** patient survival for BKPyV DNAemia.

There was no association between the risk for rejection within 3 months after kidney transplantation and the risk for BKPyV DNAemia, with an incidence of BKPyV DNAemia of 7.6% in recipients with 3-month rejection versus 6.8% in those without rejection (p = 0.740). This lack of association between rejection and BKPyV DNAemia extended to 12-month rejection, with an incidence of BKPyV DNAemia of 10.5% in recipients with 12-month rejection versus 8.3% in those without rejection (p = 0.144). There was no association between the development of new-onset donor-specific antibodies and the risk for BKPyV DNAemia (14.3% with BKPyV DNAemia versus 13.0% without, p = 0.650). We did not observe any significant difference in unadjusted death-censored graft survival when comparing BKPyV DNAemia versus no BKPyV DNAemia. A borderline difference was observed when comparing patient survival over a median follow-up of 5.3 years (see **Figures 1A, B**, respectively).

### Outcomes after BKPyVAN

There was no association between the risk for rejection within 3 months of kidney transplantation and the risk for BKPyVAN. The incidence of BKPyVAN in kidney transplant recipients with and without 3-month rejection was 9.3% and 9.1%, respectively (p = 970). This lack of association between rejection and BKPyVAN also extended to 12-month rejection, with incidences of 11.4% and 10.5%, respectively (p = 0.707).

Furthermore, there was no association between the development of new-onset donor-specific antibodies and the risk for BKPyVAN (18.2% with BKPyVAN versus 16.8% without, p = 0.704). We observed no significant difference in unadjusted death-censored graft survival or patient survival when comparing the development of BKPyVAN versus no BKPyVAN over a median follow-up of 5.3 years (see **Supplementary Figures 1A, B**).

### Systematic review and meta-analysis

**Figure 2:**
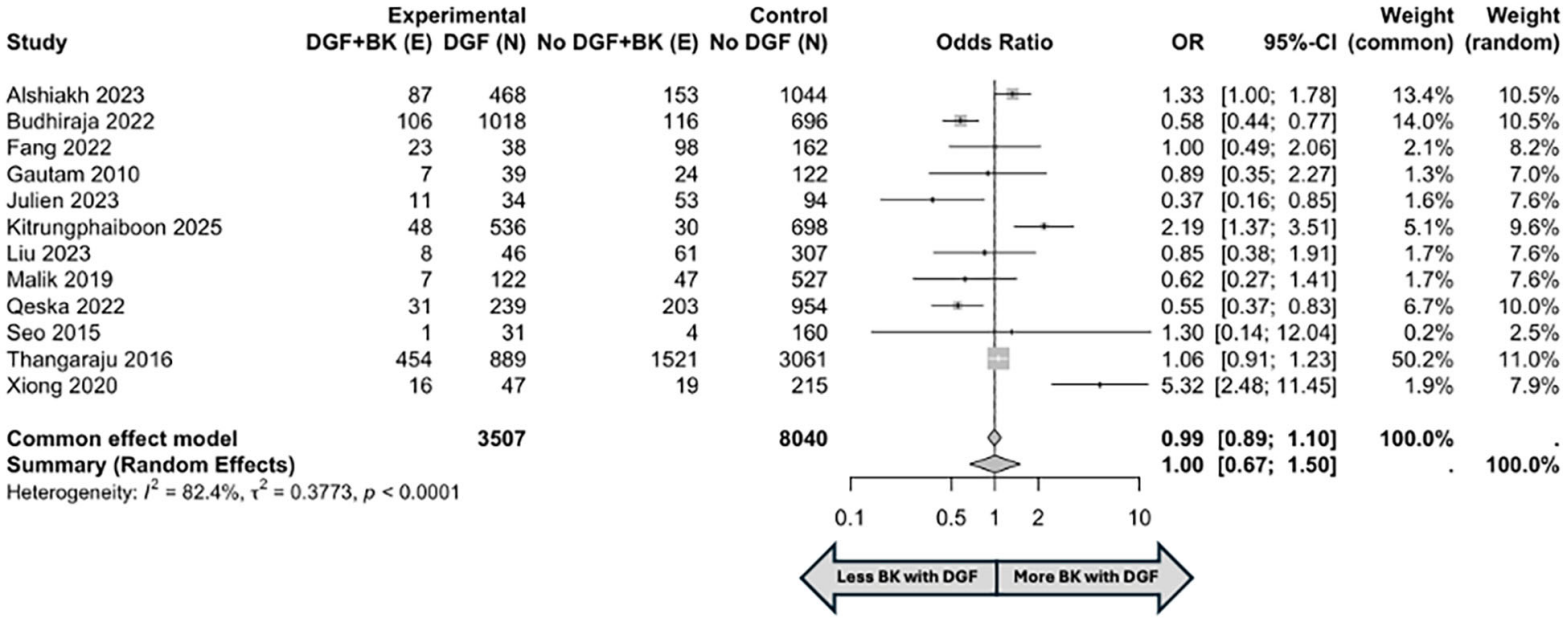
Forest plot of the studies.

In a systematic review of published literature, 5,093 studies were identified. From these, 62 reports were reviewed, and 11 studies were selected (**Supplementary Figure 2**). We combined these 11 studies with our data for empirical analysis (publication bias from these selected studies is shown in **Supplementary Figure 3**). Using a random effects model, no significant association was observed between DGF rates and BKPyV DNAemia, with an odds ratio of 1.00 (95% CI 0.67–1.50), as shown in **Figure 2**. The empirical data displayed significant heterogeneity (I2 = 82.4%). Subgroup analyses and meta-regression failed to identify underlying causes for this heterogeneity.

## Discussion

The risk of BKPyV infection remains a concern after kidney transplantation, and understanding its link with modifiable risk factors is crucial for implementing effective mitigation strategies. While a plausible pathophysiological association linking DGF and subsequent BKPyV infection has been postulated, the published literature has not found a clear connection. In our single-center analysis, although we observed a significant difference in the risk for DGF and subsequent BKPyV DNAemia in unadjusted analyses, this significance disappeared after adjusting for other BKPyV infection-associated risk factors. After combining our data with eligible studies from a systematic review, we found no evidence of an association between DGF and BKPyV DNAemia.

The published literature reports varied findings on this topic, reflected by the considerable heterogeneity observed in our empirical data. This heterogeneity stems from the diverse nature of included studies, which varied in cohort, geography, time era, and immunosuppression regimens. A further difficulty lies in the variable definitions of BKPyV DNAemia, which limits the translatability of individual study findings. For example, some studies reported routine post-transplant testing for BKPyV DNAemia (with variable frequency), some tested only by indication, and others were unclear. The latest consensus guidelines recommend surveillance testing of all kidney transplant recipients after kidney transplantation: monthly until 9 months, then 3-monthly until 2 years (or 3 years for children) (24). Greater clarity in future studies will be achieved through consistent reporting of BKPyV DNAemia after kidney transplantation, similar to the ubiquitous reporting of DGF as the need for dialysis within the first week after surgery.

The pathophysiology linking DGF and subsequent risk for BKPyV infection is putative but plausible. DGF is the immediate clinical manifestation of underlying IRI. This injury can trigger a cascade of immune activation and inflammation, contributing to endothelial dysfunction and impaired renal tubular epithelial integrity. As BKPyV primarily replicates in renal tubular epithelial cells and urothelial cells, these pathophysiologic changes and compromised renal parenchyma during DGF may provide a permissive environment for BKPyV replication by increasing cellular stress and inflammatory cytokines. DGF may also be associated with intensified immunosuppressive therapy, which further impairs antiviral immune responses, particularly T-cell-mediated control of BKPyV. Collectively, this combination of IRI, local immune dysregulation, and enhanced immunosuppression during DGF may increase susceptibility to BKPyV infection in kidney transplant recipients. However, despite this plausible pathophysiology, we did not identify any association between DGF and BKPyV infection.

The clinical implications of our findings are that patients experiencing DGF do not require more frequent BKPyV DNAemia surveillance, as our data suggest they are not at increased risk. Mandatory requirements as per international consensus guideline recommendations would be sufficient for kidney transplant recipients with DGF (24). However, it is notable that the consensus report highlighted only one risk factor as having high “grade A’” evidence for association with either BKPyV DNAemia or BKPyVAN: tacrolimus versus ciclosporin. The majority of reported risk factors had low or very low evidence (grade C or D, respectively), and DGF was not mentioned at all. There is a critical need to better understand risk factors for BKPyV infection to allow for risk stratification for surveillance and/or risk mitigation strategies. There is also no clinical justification for routine prophylactic immunosuppression modification solely based on DGF status; decision-making should be individualized and all competing risk factors weighed after kidney transplantation. Work from Eder and colleagues has shown a clear association between the risk of immunosuppression burden and BKPyVAN (35). While individualized tapering and/or reduction of immunosuppression is warranted to avoid the risk of BKPyV infection, DGF incidence should not be a deciding factor in this decision-making.

Our data did not identify an association between BKPyV infection and death-censored graft loss or mortality. This contrasts with published data suggesting that BKPyV infection (especially BKPyVAN) is associated with graft loss but not mortality. For example, Gately et al. explored outcomes after BKPyVAN using the Australia and New Zealand Data (ANZDATA) registry. They found BKPyVAN to be associated with an increased risk of all-cause (Hazard Ratio 1.75; 1.46−2.09) and death-censored graft loss (Hazard Ratio 2.49; 1.99−3.11), but not mortality (Hazard Ratio 1.15; 0.91−1.45) (19). The median follow-up in their study, at 64.8 months (± 49.2 months), was similar to that of our single-center cohort. It is unclear why death-censored graft survival outcomes differ between our center cohort and ANZDATA, but this may reflect study limitations and/or different definitions. Management of immunosuppression after BKPyVAN is also highly physician-dependent, and our single-center study lacked data on initiated changes to immunosuppression. However, data from Gately et al. highlighted that the most frequent intervention was a reduction in tacrolimus dosing by ≤50%, which occurred in 172 patients (51%). Tacrolimus was reduced by >50% in 134 patients (40%) and eliminated in 28 patients (8.4%). Mycophenolate was reduced by ≤50%, reduced by >50%, or eliminated in 134 (40%), 94 (28%), and 106 (32%) patients, respectively. It could be speculated that immunosuppression reductions were greater in the ANZDATA cohort, leading to increased DSA development and subsequent graft loss. However, the absence of reciprocal data in our cohort limits any interpretation.

Our study benefits from being a contemporary analysis of a large single-center cohort and is strengthened by its combination with published literature for a meta-analysis of all available empirical data. However, our study’s limitations must be appreciated for accurate interpretation of the data. As is typical of retrospective epidemiological analyses, there are likely confounders impacting BKPyV infection development after kidney transplantation that we were unable to factor in (e.g., donor BKPyV serostatus). Our follow-up data were only until 31 December 2020, which limits the interpretation of long-term survival data. Missing data (and misclassification bias) also affected the analyses performed, an inherent bias in epidemiological analyses such as this. We lacked data on immunosuppression changes, which limits interpretation and comparison to some published data. Some of our kidney transplant recipients were repatriated to their referral hospital, and we would not have captured their data regarding BKPyV infection. Therefore, our data may reflect an under-reporting of true BKPyV infection rates after kidney transplantation at our center. For the systematic review, significant heterogeneity in BKPyV DNAemia thresholds and immunosuppression data limited any deeper probe of the empirical data.

To conclude, our study found no association between DGF and the subsequent risk of BKPyV DNAemia or BKPyVAN in kidney transplant recipients. Further research is needed to gather more robust data on modifiable risk factors for BKPyV infection, which could facilitate risk mitigation and reduce the incidence of BKPyV infection after kidney transplantation. Future DGF studies should ensure comprehensive BKPyV infection data collection to clarify any potential association. Given the lack of clinically proven interventions, further research is required to determine the most effective strategies to reduce the risk of both DGF and BKPyV infection regardless of whether an association between the two exists to ensure long-term outcomes after kidney transplantation are optimized.

## Data Availability

The raw data supporting the conclusions of this article will be made available by the authors, without undue reservation.

## Supplementary material

Supplementary Figure 1 Unadjusted Kaplan–Meir plot of **(A)** death-censored graft survival and **(B)** patient survival for BKPyVAN.

Supplementary Figure 2 PRISMA flow diagram of the study selection.

Supplementary Figure 3 Publication bias from published studies.
