# Supplementary figures and images for: No association between delayed graft function and BK polyomavirus infection reactivation after kidney transplantation: a systematic review and meta-analysis
Source: Front Nephrol. 2026 Jun 23;6:1790723. doi: 10.3389/fneph.2026.1790723 (PMC13337812; doi:10.3389/fneph.2026.1790723)

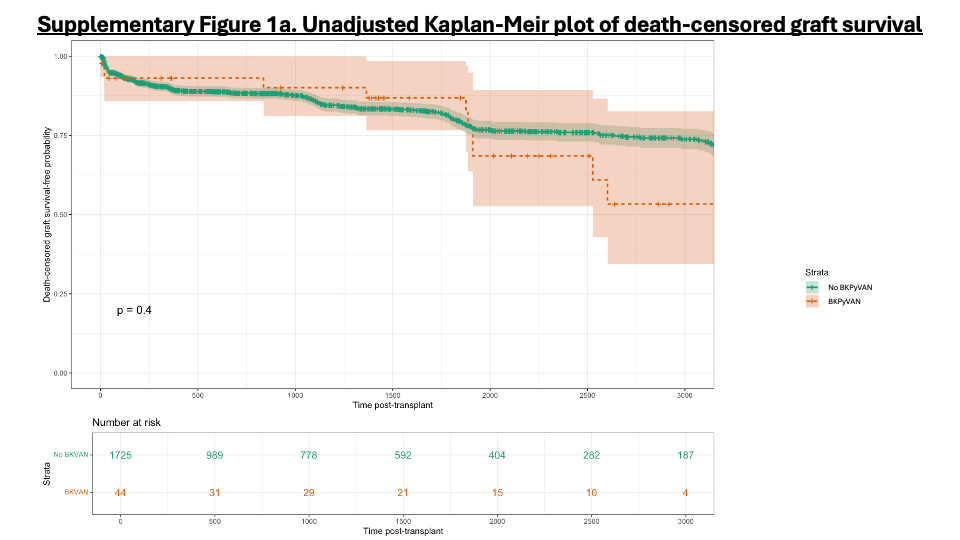

Supplement: Supplementary Figure 1 — Unadjusted Kaplan–Meir plot of (A) death-censored graft survival and (B) patient survival for BKPyVAN. [file Image1.tiff]

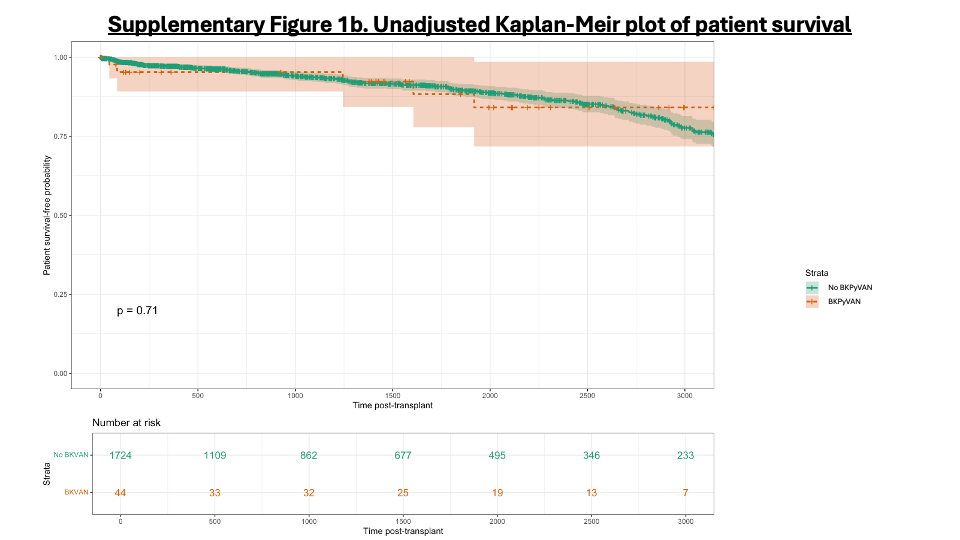

Supplement: Supplementary Figure 2 — PRISMA flow diagram of the study selection. [file Image2.tiff]

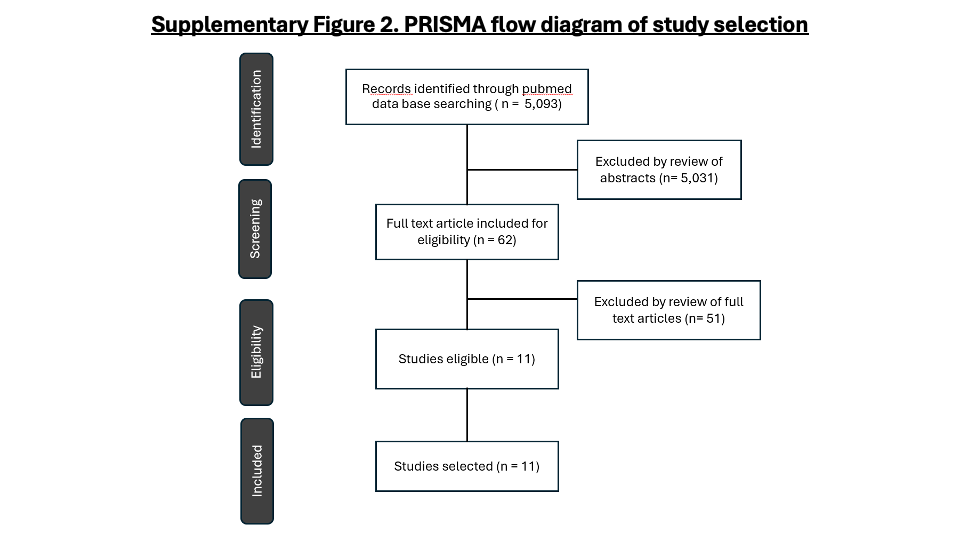

Supplement: Supplementary Figure 3 — Publication bias from published studies. [file Image3.tiff]

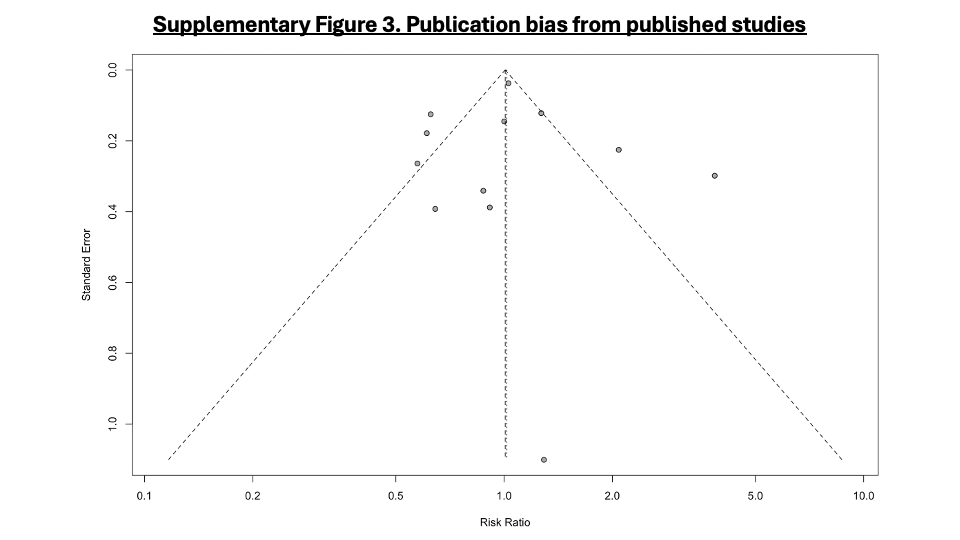

Supplement: Supplementary file 4 [file Image4.tiff]
